# Access to antimalarial drugs in the Asia–Pacific region during health emergency: a multinational cross-sectional investigation between 2020 and 2022

**DOI:** 10.1186/s41256-025-00454-6

**Published:** 2025-12-15

**Authors:** Yinuo Sun, Yuxuan Cui, Yangmu Huang, Ming Xu

**Affiliations:** Department of Global Health, School of Public Health, Peking University, No. 38, Xueyuan Rd, Beijing, 100181 China

## Abstract

**Background:**

Malaria elimination in the Asia–Pacific region has stalled in recent years, partially due to disrupted access to antimalarial drugs during public health emergencies. This study aims to explore the access to antimalarial drugs and its contextual factors in health emergencies based on investigation into six Asia–Pacific countries during the COVID-19, including Bangladesh, India, Indonesia, Pakistan, Thailand and Viet Nam.

**Methods:**

We extracted the quarterly data for 37 antimalarial drugs using the IQVIA database from the first quarter in 2020 to the second quarter in 2022. We used standard units (SU) sold per 1000 incident cases and US dollars per 1000 incident cases to evaluate consumption (accessibility). Changes in consumption were estimated using compound annual growth rate (CAGR). Associations between consumption and country’s socioeconomic, health performance and product supplier indicators were measured using least squares (pooled) panel data regression model.

**Results:**

Available antimalarial drugs ranged from 31 in India, and 6 in Bangladesh and Viet Nam. The predominant medicine category in all six countries was quinine and other quinoline derivatives. The highest level of average consumption per 1000 incident cases was observed in Viet Nam (2004141.9 SU per 1000 incident cases). The country presenting the lowest level of accessibility was Indonesia (3668 SU per 1000 incident cases). Between 2020 and 2022, all countries except Viet Nam presented a decreased consumption per 1000 incident cases, with CAGRs being respectively − 15.11% in Bangladesh, − 3.66% in India, − 23.56% in Indonesia, − 13.28% in Pakistan and − 12.07% in Thailand. Increased Log consumption per 1000 incident cases was associated with higher proportion of health expenditure out of total government expenditure (coefficient 1.84, 95% confidence interval 0.47–3.21) and higher proportion of local supply (coefficient 0.43, 95% confidence interval 0.06–0.80).

**Conclusions:**

There has been a disruption in the access to antimalarial drugs during the COVID-19 pandemic in the Asia–Pacific region, and the predominant available medicines were those with documented resistance. Greater priority should be given to drug innovation aimed at improving availability, along with strengthening health systems and local production to maintain accessibility to these drugs, especially during health emergencies.

**Supplementary Information:**

The online version contains supplementary material available at 10.1186/s41256-025-00454-6.

## Introduction

As one of the major public health concerns, malaria cases increased between 2020 and 2023 [1–3]. The number of malaria cases was estimated at 263 million in 2023, with an increase of 11 million cases from 2022 [1]. Though some countries have moved to malaria elimination phases, malaria continues to pose a major threat to the populations, especially in African countries followed by Asia–Pacific countries [1, 4]. Only Sri Lanka and China in the Asia–Pacific region have achieved malaria elimination. According to the WHO report 2024, the South-East Asia and Western-Pacific regions respectively account for 4 and 1.7 million cases in 2023 [1]. An additional estimated 1.72 billion people are at risk of developing malarial infection in the Asia–Pacific region [5, 6]. Many countries in the Asia–Pacific region face stagnated progress and have far way to reach the malaria elimination goal before 2023, including India, Pakistan, Indonesia, Myanmar, Bangladesh, Thailand, Philippines, and Viet Nam [5].

Antimalarial drugs offer the opportunity to prevent most deaths and severe complications through timely and effective treatment, making malaria a curable condition. The range of antimalarial drugs developed began with the discovery of quinine in the early 1800s and continued through to modern-day recommended artemisinin-based combination therapies (ACTs) in the 2000s [7–9]. Quinine-based therapies were recommended for *P. vivax malaria,* and ACTs are recommended by WHO as the first- and second-line treatment for *P. falciparum* malaria [9]. Currently recommended available alternatives include artemisinin-based combination therapies (ACTs), artemisinin and its derivatives (ARTs) (e.g., artesunate), quinine and other quinoline derivatives (e.g., chloroquine), and quinine-based combination therapy [7, 10].

However, despite the increasing incident cases, recent health emergencies triggered by emerging infectious diseases, climate change, and armed conflict have disrupted healthcare supply chains, resulting in critical disruptions to malaria drugs [11]. Disrupted access to malaria drugs heightens transmission risk and mortality in endemic regions [1]. The WHO has prioritized that the impacts of COVID-19 have caused malaria treatment disruption in an estimated 58 endemic countries [12]. Other health emergencies such as catastrophic flooding in Pakistan have further complicated the challenges in access to antimalarial drugs. Understanding the systemic effect of the pandemic on overall use and its influencing factors is important for maintaining the access to medicine during health emergencies [13].

Existing studies have predominantly focused on overall disease burden [14], diseases control [15] or drug efficacy [16], with limited analysis on the accessibility of specific antimalarial drugs. There are also few findings on the effects of COVID-19 on malaria control in different regions. In this study, we analyze the availability and accessibility of antimalarial drugs in six countries and the disruption during the COVID-19 pandemic. We also explored the impact of health, economic and industry performance indicators on antimalarial drugs consumption. Our findings hope to identify potential factors and methods to strengthen the resilience of the antimalarial drug supply chain in health emergencies and to inform antimalarial global action in response to possible future outbreaks.

## Methods

### Study setting

We included six Asia–Pacific countries in our study based on the following criteria: First, we identified the Asia–Pacific countries where malaria elimination has yet to be achieved. Second, we choose countries with varying disease burden, health system capacity and income level to make comparisons more meaningful. Third, we included countries whose data were available in IQVIA-Multinational Integrated Data Analysis System (MIDAS) database. Bangladesh, India, Indonesia, Pakistan, Thailand, Viet Nam were finally selected to be included in our study. The characteristics of these six countries are presented in Table 1 in Additional file 1.

### Data source

We extracted quarterly data on sale volumes in standard units (SU) and manufacturer level sales in US dollars of all antimalarial drugs from 2020 Q1 to 2022 Q2 from the IQVIA database. The proprietary dataset captures medicines sales volumes and values from pharmacies, wholesalers, distributors and manufacturers in the hospital and retail sectors in 93 countries, which are extrapolated to represent national-level sales [17–19]. The accuracy and representativeness of the data have been checked and ensured through specifically developed algorithms [18]. The data set encompassed country, setting (retail or hospital), generic name, quarter, year, strength, formulation, manufacturer name, sale volumes in standard units (SU), and sale values in United States dollars (USD). A SU refers to the smallest common dose of a product form and it facilitates the comparison of antimalarial drugs in different forms across various countries.

Availability. All antimalarial drugs listed in section “P1D anti-malarials” were included in our analysis. We further classified the included 37 medicines into six categories based on the active ingredients, namely quinine and other quinoline derivatives, quinine-based combination therapy, artemisinin and its derivatives (ARTs), artemisinin-based combination therapies (ACTs), other chemical medicines and traditional Indian ayurvedic medicine.

Accessibility (Consumption). We considered that the sales volumes extracted from IQVIA-MIDAS were equal to the consumption in each quarter of antimalarial drugs across included countries [18–21]. To evaluate the available products allocated to malaria cases, we further calculated the number of SUs sold per 1000 incident cases in a population. The incident case data for each country from 2020 to 2022 were derived from the World Health Organization Malaria Report 2023.

Health-social-economic factors. Previous studies have included key Health-social-economic factors for drug accessibility, such as health expenditure proportion, Universal Health Coverage service coverage index (UHC SCI), life expectancy, Gross Domestic Product (GDP) per capita, and the Human Development Index (HDI) [19, 22]. Clinical literature does suggest a link between diabetes and cardiovascular disease and severity of malaria cases [23, 24]. In addition, these two indicators were often adopted as proxies for the health system capacity. Therefore, we selected following health-social-economic factors for each country: (1) malaria incidence rate calculated according to WHO malaria report 2023; (2) prevalence of cardiovascular diseases; (3) prevalence of diabetes diseases; (4) percentage of health expenditure out of total government expenditure; (5) UHC service coverage index; (6) life expectancy at birth; (7) GDP per capita; (8) Human Development Index; (9) different role of health systems through identifying the responsible sectors for health regulation, financing and service provision as private (India, Viet Nam, and Indonesia) and public (Bangladesh, Pakistan and Thailand)] [25–29]. The data were collected from international agencies, including the IHME, World Health organization, World Bank and Our World in Data, using data from the corresponding year. In addition, the localized production capacity of pharmaceuticals directly impacts their local supply. We further investigate its impact on treatment accessibility. Following the previous studies, we classified the manufacturer of included medicine into local and abroad ones, and calculated the quarterly proportion of local supply volume. We considered the local manufactures if they were online reported as a domestic source.

### Data analysis

Consumption was summarized using the mean between 2020 Q1 and 2022 Q2. The compound annual growth rates (CAGR) was calculated to evaluate the trend in the consumption from 2020 Q1 to 2022 Q2 for each country. The specific equation was as follows:

$${\text{CAGR}}_{{{\text{consumption}}}} = \left[ {\left( {\frac{{{\text{SU}}\,{\text{per}}\,{\text{year}}\,{\text{per}}\,{\text{thousand}}\,{\text{cases}}\,{\text{in}}\,{2022}\,{\text{Q2}}}}{{{\text{SU}}\,{\text{per}}\,{\text{year}}\,{\text{per}}\,{\text{thousand}}\,{\text{cases}}\,{\text{in}}\,{2020}\,{\text{Q1}}}}} \right)^{\frac{1}{10}} - 1} \right]$$

We then conducted 3 pooled panel regression models to examine the associations between log quarterly antimalarial drugs consumption per 1000 incident cases and these health, economic and system indicators. The first model has all variables excluding GDP, HDI and life expectancy at birth. The second model has all variables excluding life expectancy at birth, and the third model included all the above variables. We considered *p* < 0.05 for statistical significance. STATA 16.1 was used for analysis.

## Results

### Availability of antimalarial drugs during 2020–2022

**Fig. 1:**
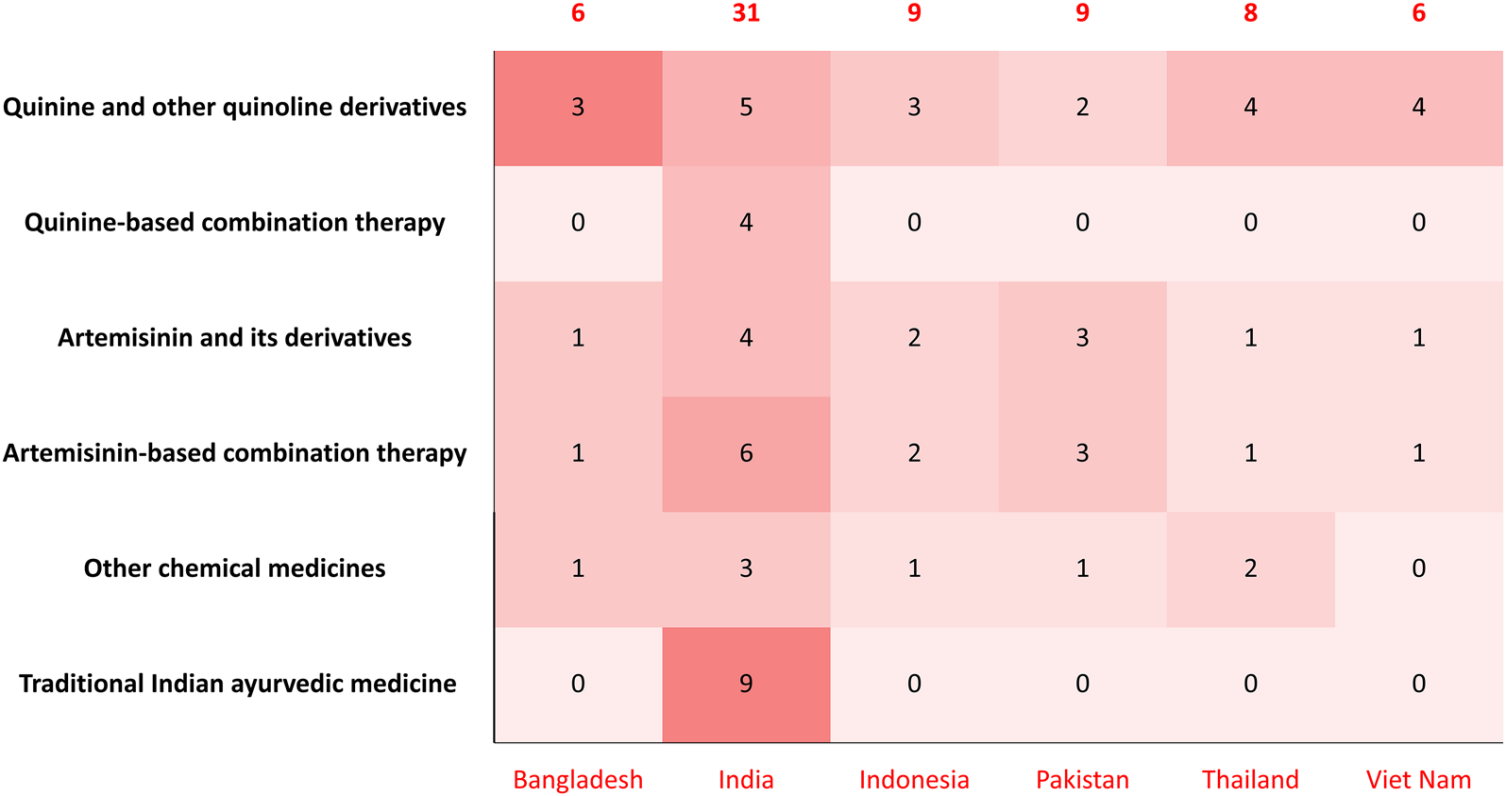
Number of available antimalarial drugs by country between 2020 and 2022

We analyzed the available antimalarial products in the included countries. Available antimalarial drugs ranged from 31 in India to 6 in Bangladesh and Viet Nam (Fig. 1). Quinine and other quinoline derivatives, artemisinin and its derivatives (ARTs), and artemisinin-based combination therapies (ACTs) were available in all six countries. India had the highest number of anti-malaria drugs in terms of all five sets of available products. Quinine-based combination therapy and traditional herbal medicine were only available in India.

### Accessibility of antimalarial drugs, 2020–2022

**Fig. 2:**
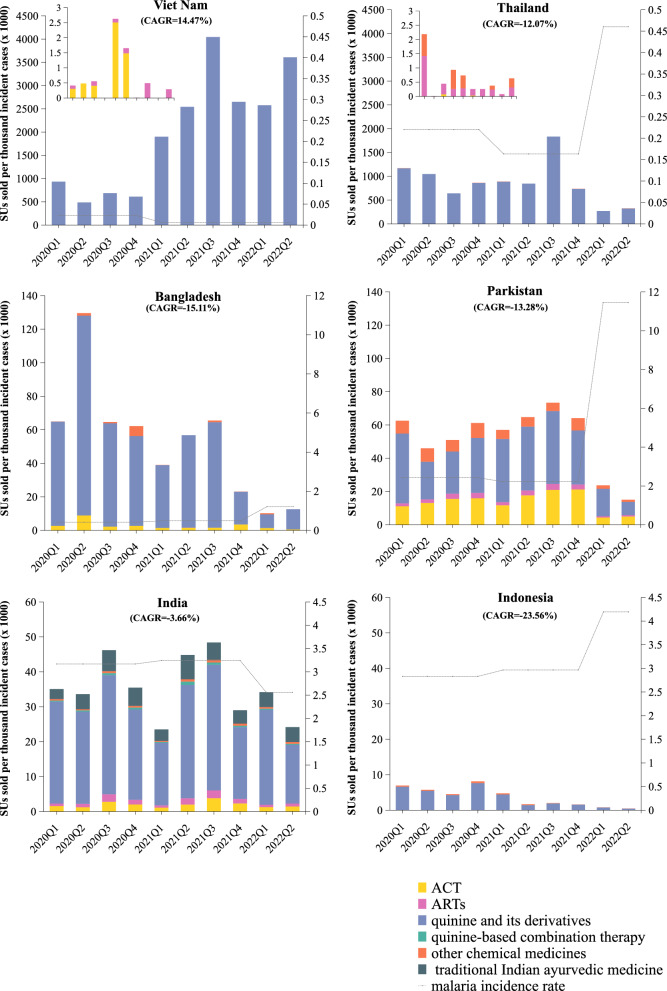
Quarterly antimalarial drugs accessibility (SU/1000 incident cases) by country and medicine between 2020 and 2022

The total SU consumption from 2020 to 2022 of anti-malaria drugs ranged from 459,330.2 SU in Bangladesh to 144,092,221 SU in India (Table 2 in Additional file 1). We also examined the consumption by drug types. The highest total consumption was observed in quinine and other quinoline derivatives in all six countries. The proportion of quinine and other quinoline derivatives SU consumption accounted for more than 90% in Viet Nam (99.95%), Thailand (99.92%), Indonesia (94.10%) and Bangladesh (92.80%) (Table 2 in Additional file 1). The consumption per 1000 incident cases of anti-malaria drugs was imbalanced across the selected Asia–Pacific countries. The highest level of average consumption per 1000 incident cases was observed in Viet Nam (2004141.9 SU per 1000 incident cases). The country presenting the lowest level of accessibility was Indonesia (3668 SU per 1000 incident cases) (Fig. 2).

Figure 2 shows the country-specific sales volume per 1000 incident cases by medicine types, and the corresponding CAGRs of consumption per 1000 incident cases in each country. Between 2020 and 2022, the consumption per 1000 incident cases showed a decreasing trend in Bangladesh (CAGR = − 15.11%), India (CAGR = − 3.66%), Indonesia (CAGR = − 23.56%), Pakistan (CAGR = − 13.28%), Thailand (CAGR = − 12.07%). Viet Nam was the only country which held an increasing consumption per 1000 incident cases between 2020 and 2022 from 933,847.3 SU per 1000 incident cases in 2020 Q1 to 3,608,881.068 SU per 1000 incident cases in 2022 (CAGR = 14.47%).

### Association between access to antimalarial drugs and health, economic and system factors

Tabl﻿e 1 presents the pooled (model 1, model 2 and model 3) regression results. We found that incidence of malaria was negatively associated with the consumption (− 0.08, *p* < 0.05 in model 1; − 0.09, *p* < 0.05 in model 2; − 0.05, *p* < 0.05 in model 3), confirming the existence of supply disruptions during the pandemic. The mitigating factors are then the proportion of local supply and health expenditures as % of government expenditure. In model 3, holding other factors constant, a 10% relative increase in the proportion of local supply is associated with a 4.3% increase in consumption per 1000 incident cases. Similarly, an increase in percentage of health expenditure out of total government expenditure by 10% is associated with 18.4% increase in the consumption per 1000 incident cases.

**Table 1:** Least squares (pooled) panel data regression analysis of factors associated with log anti-malaria consumption per 1000 incident cases: 2020Q1–2022Q2

Factor	Model 1, coefficient (95% CI)	Model 2, coefficient (95% CI)	Model 3, coefficient (95% CI)
Malaria incidence rate (per 100,000 people)	− 0.08 (− 0.13, − 0.03)*	− 0.09 (− 0.15, − 0.03)*	− 0.05 (− 0.08, − 0.02)*
Proportion of local supply (%)	− 1.73 (− 3.90, 0.43)	− 1.20 (− 4.08, 1.68)	0.43 (0.06, 0.80)*
Health system (public or private)	− 0.89 (− 2.93, 1.14)	− 1.82 (− 5.11, 1.47)	− 2.12 (− 5.67, 1.43)
Prevalence of cardiovascular diseases (per 100,000 people)	− 0.0003 (− 0.0006, 0.0000)	− 0.0002 (− 0.0007, 0.0003)	0.0004 (− 0.00005, 0.0008)
Prevalence of diabetes diseases (per 100,000 people)	− 0.002 (− 0.004, 0.001)	− 0.002 (− 0.005, 0.001)	0.002 (− 0.001, 0.004)
Percentage of health expenditure out of total government expenditure (%)	− 0.35 (− 0.91, 0.21)	− 0.40 (− 1.03, 0.24)	1.84 (0.47, 3.21)*
UHC service coverage index	0.28 (− 0.05, 0.62)	0.39 (− 0.06, 0.83)	0.07 (− 0.34, 0.48)
Life expectancy at birth		− 0.21 (− 0.80, 0.38)	− 0.50 (− 1.14, 0.15)
Human Development Index			36.13 (− 25.20, 97.46)
Gross domestic product per capita (USD per capita)			− 0.002 (− 0.004, 0.000)

The outcome variable of the regression is log anti-malaria consumption per 1000 incident cases. *p < 0.05

## Discussion

Access to antimalarial drugs in the Asia–Pacific region continues to be a major challenge. This study used pharmaceutical sales data from six countries and applied panel data regression models to explore the disruption and its influencing factors from the lens of health, economic and industry performance during pandemic. There were limited drug options available in most Asia–Pacific countries. All countries except Viet Nam have witnessed a disruption to antimalarial drugs during COVID-19. The accessibility of antimalarial drugs was positively associated with percentage of health expenditure out of total government expenditure and the level of localized production. Strengthening national health spending and localized production levels can help to increase access to antimalarial drugs and improve the robustness and resilience of the antimalarial supply chain to cope with future outbreaks.

The pattern of available antimalarial drugs in the Asia–Pacific region was highly different. The number of available medicines was no more than ten in Bangladesh, Indonesia, Pakistan, Thailand, Viet Nam, which was mainly related to the innovative capacity in these countries [30]. Quinine and other quinoline derivatives were the most widely available antimalarial agents in this region. In countries such as Viet Nam, Laos, and Cambodia, *p. vivax* infections predominated, for which quinine-based therapy remained the recommended treatment. However, malaria epidemiology grew increasingly heterogeneous across most countries, marked by rising Plasmodium falciparum infections, particularly in Myanmar, Pakistan, and India. Although ACTs are endorsed as first-line treatments for *P. falciparum* malaria, access to these medicines remains constrained in resource-limited settings, largely due to inadequate availability and affordability of ACTs [31]. Compounding this challenge, resistance to conventional antimalarials, including quinine, chloroquine, and primaquine, has been documented [32–34], alongside emerging and spreading resistance to artemisinin and its partner drugs across the Asia–Pacific region [35]. Therefore, the control of malaria in this region was faced by a double challenge of access to antimalarial medicine and drug resistance. We observed that India has included traditional Indian ayurvedic medicine into malaria control. It has long been used in India as a complement in areas where the access to modern medicine was limited. The introduction of other potentially desirable therapies, such as traditional herbal medicines, triple artemisinin-based combination therapy (TACT) and single-encounter radical cure and prophylaxis (SERCaP), may yield even greater benefits, but there is a lack of sufficient clinical data to support this [36–38]. Notably, the development of SERCaP may more hinder than help the urgently needed clinical development of new anti-malarial drug regimens [39]. In the future, promoting diversified drug innovation should be highly prioritized. This strategy should ensure both therapeutic accessibility and sustainable resource allocation, avoiding overcommitment to a single high-risk target that may undermine the development of broader anti-resistance tools.

Maintaining the accessibility is essential for malaria treatment, especially in the health emergency. The COVID-19 pandemic has exacerbated weaknesses and disparities in the supply chain for antimalarial drugs. Our study shows an overall downward trend in the volume and sales of antimalarial drugs in five Asia–Pacific countries except Viet Nam during COVID-19, which echoes the rise in malaria incidence and deaths in the Asia–Pacific region between 2020 and 2022 [3]. Data from the first round of the WHO PULSE survey show that basic health services related to infectious diseases are, on average, moderately affected by pandemics across the Asia–Pacific region [40]. The latest data from a geospatial modelling analysis show a 25% reduction in antimalarial drugs coverage in African countries in 2020, which is in line with the trend of our findings [41].

The association analysis showed that country-specific levels of health expenditures and localized production are mitigating factors for the estimated associations between malaria cases and antimalarial drugs consumption. We acknowledge that there are other factors which may have not been fully considered in the current analysis. As reported in previous studies, percentage of health expenditure out of total government expenditure is one of the most significant drivers of treatment access, which was aligned with our finding [22, 42, 43]. While private and public system differences were not significant in our analysis, prior evidence links private sector dominance to lower drugs accessibility. Strategic government investments in health, notably in health workforce, appear pivotal. Viet Nam's deployment of community malaria workers secured drug access during COVID-19 [44]. However, consistent with established literature, Indonesia's supply decline reflected healthcare access barriers, including both health workers and facilities [45].

Our study also revealed that the level of localized production is an important influence on the accessibility of antimalarial drugs during a pandemic, consistent with statements and initiatives by WHO [46]. Some countries face problems in accessing medicines due to low manufacturing capacity and that such problems can be exacerbated in times of health emergencies and/or overwhelming demand [47]. COVID-19 further highlights the importance of  localized production in maintaining the supply of medicinal products during a pandemic. The global embargo and severe travel restrictions brought about by the pandemic have disrupted the global drug manufacturing and trading system, with many countries increasing their stockpiles of essential medicines and reducing exports of raw materials and finished products [48]. Several countries in the Asia–Pacific region were facilitated with high manufacture capacity on antimalarial drugs, including China and India [49]. Conversely, countries in the Asia–Pacific region—including Bangladesh, Laos, and Indonesia—are heavily dependent on imports of antimalarial drugs [50]. Collective efforts and commitments should be encouraged to enhance localized production through business ecosystem building, multi-stakeholder collaboration, capacity building, training, regional/global approaches, et al., to promote greater sustainability of supply chains.

This study has several limitations. First, we used data from a combination of public and commercial databases. While these estimates are extrapolated from the most reliable sources available, nuances in the estimation of identical indicators across various datasets and data quality issues may result in inaccurate assessments. Second, we did not include all the malaria endemic countries in the Asia–Pacific region due to limited access to the IQVIA database. Our study selected six representative Asia–Pacific countries with varying disease burdens, income levels, and health system performance. Third, the consumption was underestimated by using sales volume data, which may not account for informal or unreported channels. Additionally, the potential delays between sales and usage could obscure the temporal relationship between reported sales and true consumption. Fourth, our analysis didn’t allow one to assess pre- and post-pandemic consumption patterns. The paper only showed reduced antimalarial use during COVID-19 in most countries, but without a counterfactual, one cannot tell whether the same sort of supply disruptions would have occurred with or without COVID-19. Finally, we acknowledge that the association analysis conducted does not imply causality and other factors that may correlate with health access may not be fully considered and adjusted in the current analysis. Further research is recommended to explore the underlying mechanisms of how these factors interact. In addition, further qualitative studies exploring patient and provider perspectives would provide valuable depth to the findings.

## Conclusion

In the six Asia–Pacific countries, the availability of antimalarial drugs remains insufficient and the predominant categories were those confirmed with drug resistance. In addition, the accessibility of antimalarial drugs experienced a disruption during the COVID-19 pandemic. We observe significant associations between the accessibility of antimalarial drugs and  both the proportion of health expenditure and local supply of drugs. Increasing national investment in the health system and strengthening local production could help ensure the accessibility especially during health emergency. Apart from maintaining accessibility, innovation and research should be prioritized to improve the availability of antimalarial drugs.

## Supplementary Information

**Additional file 1**.

## Data Availability

The data used in this study are from public and proprietary databases.

## Abbreviations

UHC SCI: Universal Health Coverage service coverage index
HDI: Human development index
GDP: Gross domestic product
WHO: World Health Organization
CAGR: Compound annual growth rate
TACT: Triple artemisinin-based combination therapy
SERCaP: Single-encounter radical cure and prophylaxis
ACTs: Artemisinin-based combination therapies
ARTs: Artemisinin and its derivatives
SU: Standard units
