# Supplementary material for: Access to antimalarial drugs in the Asia–Pacific region during health emergency: a multinational cross-sectional investigation between 2020 and 2022
Source: Glob Health Res Policy. 2025 Dec 15;10:66. doi: 10.1186/s41256-025-00454-6 (PMC12703887; doi:10.1186/s41256-025-00454-6)
Supplement: Supplementary file 1 — Additional file 1. [file 41256_2025_454_MOESM1_ESM.pdf]

Supplementary Table 1 Characteristic of the 6 Asia-Pacific countries included in this study

	Bangladesh	India	Indonesia	Pakistan	Thailand	Viet Nam
GDP per capita in 2022 (USD per capita)	2716.5	2391.9	4798.1	1650.3	7069.6	4086.5
Malaria incidence cases in 2022	22451	3389400	1155531	2655017	6263	412
percentage of health expenditure out of total government expenditure in 2022 (%)	1.19	4.46	8.03	5.55	16.10	10.70
universal health coverage index	52	63	55	45	82	68
Human development index in 2021	0.67	0.644	0.713	0.54	0.803	0.726
life expectancy at birth in 2022 (years)	74	68	68	66	80	75

Supplementary Table 2 The total SU consumption by drug categories in6 Asia-Pacific countries

	**Bangladesh**	**India**	**Indonesia**	**Pakistan**	**Thailand**	**Viet Nam**
**Total SU consumption**	4593302	1440922210	2881844420	5763688840	11527377680	14270231
Artemisinin-based combination therapy	245256	80673732	1521595942	4403440362	10167129202	5408
Artemisinin and its derivatives	940	52106361	52106361	52106361	52106361	1223
Quinine and other quinoline derivatives	4260386	1083029947	1083029947	1083029947	1083029947	14263600
Quinine-based combination therapy	0	18580022	18580022	18580022	18580022	0
Other chemical medicines	86720	20363404	20363404	20363404	20363404	0
Traditional Indian ayurvedic medicine	0	186168744	186168744	186168744	186168744	0
